# The Neural Correlates of Emotional Prosody Comprehension: Disentangling Simple from Complex Emotion

**DOI:** 10.1371/journal.pone.0028701

**Published:** 2011-12-12

**Authors:** Lucy Alba-Ferrara, Markus Hausmann, Rachel L. Mitchell, Susanne Weis

**Affiliations:** 1 Department of Psychiatry and Neuroscience, University of South Florida, Tampa, Florida, United States of America; 2 Department of Psychology, Durham University, Durham, United Kingdom; Cuban Neuroscience Center, Cuba

## Abstract

**Background:**

Emotional prosody comprehension (EPC), the ability to interpret another person's feelings by listening to their tone of voice, is crucial for effective social communication. Previous studies assessing the neural correlates of EPC have found inconsistent results, particularly regarding the involvement of the medial prefrontal cortex (mPFC). It remained unclear whether the involvement of the mPFC is linked to an increased demand in socio-cognitive components of EPC such as mental state attribution and if basic perceptual processing of EPC can be performed without the contribution of this region.

**Methods:**

fMRI was used to delineate neural activity during the perception of prosodic stimuli conveying simple and complex emotion. Emotional trials in general, as compared to neutral ones, activated a network comprising temporal and lateral frontal brain regions, while complex emotion trials specifically showed an additional involvement of the mPFC, premotor cortex, frontal operculum and left insula.

**Conclusion:**

These results indicate that the mPFC and premotor areas might be associated, but are not crucial to EPC. However, the mPFC supports socio-cognitive skills necessary to interpret complex emotion such as inferring mental states. Additionally, the premotor cortex involvement may reflect the participation of the mirror neuron system for prosody processing particularly of complex emotion.

## Introduction

Human everyday verbal communication involves not only semantic but also non-linguistic, information being carried by the voice [1]. This phenomenon, known as prosody, comprises acoustic features such as pitch, amplitude, segment and pause duration and allows for the encoding and decoding of emotions in speech [1], a skill which is necessary to ensure effective social communication [2].

We will denote the act of decoding emotion cues conveyed by prosody as emotional prosody comprehension (EPC). EPC does not represent a single construct. There are qualitative differences between simple emotions and more complex emotional states. Therefore, EPC is a multi-level mechanism, from the decoding of simple emotions such as fear, happiness or anger to the assessment of complex mental states. Furthermore, EPC is regarded as one of the precursor of emotional theory of mind [3]. Evolutionary, simple emotions evolved for “their adaptive value in dealing with fundamental life tasks” [4]. They are shared with other primates, include a distinctive, universal physiological response [4] and are characterized by automated and complex changes involving facial and vocal expressions [5]. They only last for a limited period of time, are highly stereotypical and involve very limited cognitive processing [5]. In contrast, complex emotions, and especially social emotions such as pride, guilt and embarrassment, require the interpretation of social intentions [6], consideration of other people, comprehension of social norms and recognition of personal responsibility for the consequences of a situation [7]. They require the monitoring of attitudes and opinions of others regarding our own behaviour, are culturally dependent, and rely upon the evaluation of others [8]. Non-social complex emotions, such as thoughtfulness, boredom or interest are belief-based rather than situation-based and reflect the inner thoughts of an individual [9]. An important difference between complex emotion and simple emotion is based on the fact that complex emotions involve adjudicating a cognitive state as well as an emotion and are context and culture dependent [10] [11]. The cognitive content is an essential constituent of the emotion and it is a relevant part of what causes the emotion [12]. Thus, complex emotions are a cognitively enriched extension of simple emotion [12] and additional cognitive elaboration is necessary to process complex mental states [13].

At the behavioural level, studies have sought to determine whether emotion comprehension for simple emotion and complex emotion is differentially affected by neurological impairments and childhood development, which might imply separate neural processes. However, findings have been equivocal. One study in children with learning disabilities revealed difficulties in understanding complex social emotions such as pride or guilt together with a preserved ability to recognize simple emotions such as happiness or sadness [7], suggesting that both emotional processes might be neuronally dissociated. In agreement with these finding, another study using facial stimuli found a similar dissociation between the comprehension of simple and complex emotion in patients with schizophrenia [14]. On the other hand, a recent study looking at the detection of sarcasm (a complex emotion) and simple emotion from vocal cues found that performances in both tasks were highly correlated in both a control and a schizophrenic patients group. [15].

Looking at functional brain imaging data, some clinical studies have indicated overlapping brain areas involved with simple and complex emotion comprehension deficits [16]–[17]. To the best of our knowledge, however, there have not yet been any studies in healthy participants which have directly compared the brain networks involved with simple and complex emotion comprehension from speech cues. Such studies might be especially interesting, as simple and complex emotion comprehension might be mediated by different brain areas, even if they appear correlated on the behavioural level. Previous neuroimaging studies have shown that, EPC in general is supported by a temporo-frontal network [18]–[21]. However, the role of each of the neural components in the network, particularly the involvement of prefrontal nodes, is still under debate.

Some authors have claimed that the involvement of prefrontal regions in EPC depends on linguistic features of the stimuli. One study [22] found that concurrent semantic content of prosodic cues resulted in increased activation of the inferior frontal gyrus, while activation of the posterior lateral temporal lobe during prosody decoding remained constant independently of the semantic load of the stimuli [21]. Interestingly, it has been suggested that increased (semantic) processing demands may therefore have little effect on the auditory cortex response, but may modulate the frontal lobe response [23]. Conversely, it has been proposed that taking away the labelling element of typical EPC tasks (such as classifying the stimuli into a category represented with a word like “happy”) and asking participants to discriminate EPC instead (make same/different judgements about the emotion conveyed in pairs of sentences) reduces frontal lobe activity [23], indicating that the demand on frontal lobe resources is reduced when EPC are reduced to purely perceptual judgements.

An alternative model of emotion perception proposes that in order to decode other persons's emotions, postural, facial or vocal cues are observed, which activate engrams to simulate a similar emotion [24]. Such an internal simulation facilitates the sensation of the emotional state in an embodied way, which then is interpreted and attributed to other individuals. If this model is correct, the recruitment of a mirror neuron system for the perception of emotions would be necessary. In fact, the role of a mirror system for emotion decoding from facial emotion [25]–[26] as well as prosody [27]–[29] have been proposed. Some of these studies suggested that the engagement of the mirror system depends on the empathic characteristic of the participants [27]. For example, in a prosody decoding task, activations in the bilateral superior, middle and inferior frontal gyri, as well as the anterior insula and bilateral perisylvian activation inversely correlated with empathic ability [27]. The extensive activation including the bilateral superior, middle and inferior frontal gyri may relate to inner simulation of the emotional state of others [30] which might be particularly crucial for more demanding emotions in which the inference of intentions is required [31]. Thus, it is plausible to predict that premotor activation would be more prominent for complex social emotions in comparison to simple.

The mirror neuron system helps to understand the mental states of other on the basis of our own mental state, which is the first step for theory of mind (ToM) [32]. It has been proposed that making inferences about social interactions (a task which requires ToM) relies upon the integrity of the orbitofrontal as well as the medial prefrontal cortex [32]–[33]. ToM skills may be particularly needed in EPC for complex and social emotion because they imply to adjudicate inner thoughts to the individual experiencing the mental state in the case of the non-social complex emotion, whereas social emotion require the interpretation of social cues, taking the dyadic relation in which the emotion emerges. If this is true, the neural network underlying ToM should also underlie EPC for complex and social emotions.

The present study examines the neural correlates of EPC of simple and complex emotion from vocal cues. This investigation proposes that EPC for both simple and complex emotion share common neural components, but additional socio-cognitive modules are recruited for complex emotion. It is hypothesised that the neural correlates of the complex emotion comprehension differ from those of simple emotion due to the requirement of taking the emotional perspective of other [33] which might partly rely on mental state decoding skills [34]. Specifically, we predict that EPC for complex emotion involves activation of the orbitofrontal and medial PFC as part of the social brain [35] as well as the premotor cortex as a part of the mirror neuron system, indicating that the involvement of the PFC in EPC depend on the complexity of social judgments involved in the task.

## Methods

### Participants

A group of twenty male students and academics staff were recruited from the Department of Psychology at Durham University. Only male participants were recruited because women present larger variability in functional brain organization, partly due to hormonal fluctuations across the menstrual cycle [36]–[37]. Also, emotional state is affected hormonally, for example, across the menstrual cycle [38], causing unwanted additional variability. Students received course credits for taking part in the study. One participant had to be excluded from the analysis due to artefacts caused by an orthodontic brace. The mean age of the remaining 19 male participants was 24.8 years (*SD* = 8.79 years, age range: 18 to 51 years). All participants were native English speakers and reported not to have any history of psychiatric disorders, hearing impairment, history of drug or alcohol abuse, long periods of unconsciousness or head injuries. Ethical approval for the study was obtained from the Ethics Sub-Committee of the Psychology Department at Durham University.

### Stimuli and task

The EPC stimuli used in the current study were selected from Banse and Scherer's study of vocal emotion expression [39] and comprised numbers spoken in different tones of voice. Stimuli were created by the Linguistic Data Consortium (LDC), an open consortium of universities, companies and government research laboratories, hosted by the University of Pennsylvania. The utterances were recorded by six professional actors (three male, three female) recorded on two channels, with a sampling rate of 22.05K, and two microphones, a stand-mounted boom Shure SN94 and a headset Sennheiser HMD 410. Sound files were encoded in interleaved 16-bit PCM, high-byte-first format. Further details about the EPC stimuli can be found at http://www.ldc.upenn.edu.

From the full set of numbers, stimuli spoken in three simple emotional tones (happy, sad, angry) and three complex/social tones (proud, guilty, bored) were selected. Simple and complex emotion stimuli were presented in two separate functional MRI runs, administered in a counterbalanced order across participants. For both conditions, also numbers spoken in a neutral tone of voice and silent trials (baseline condition) were employed, resulting in a total of 160 utterances (32 per emotional category, 32 neutral stimuli and 32 silent trials per run). The mean duration of each sound stimulus was 2010 ms (range: 1800 to 2080 ms). Stimuli were presented using E-Prime (Psychology Software Tools, Philadelphia) via Phillips digital stereo headphones. The order of trials was selected based on statistical efficiency computations by an fMRI simulator software taking into account the shape and timing of the canonical hemodynamic response function employed for modeling the time course of the BOLD response in SPM. More details about this software can be found at http://www.cabiatl.com/CABI/resources/fmrisim/.

In both runs, participants were asked to classify the emotion conveyed by the tone of voice for each trial in one of the emotional categories and to indicate their forced-choice response by pressing one of four keys (one for each emotional valence) of a five key response box using the right hand. A picture of the response box indicating which key corresponded to which response was continuously presented on the screen. Participants were asked to respond as fast and as accurate as possible.

Even though the task used in the present study requires the categorization of emotions, the use of the term emotional prosody comprehension (EPC) is more in line with the literature. A vast amount of prosody studies applying similar tasks to the present investigation referred to emotional prosody comprehension [2], [40]–[43].

### Image Acquisition

Functional MRI images were acquired with a Phillips Achieva 3T scanner with a SENSE standard 8-channel birdcage head coil. The functional gradient-echo echoplanar T2*-weighted images (EPI) were acquired with an echo time (TE) of 30 ms, a flip angle of 90°, a field of view (FOV) of 192 mm and an in plane resolution of 64×64 voxels. Each functional image consisted of 28 axial slices (4 mm thickness with 0.5 gap), which covered the whole cerebral cortex. In order for participants to be able to hear the auditory stimuli during the functional runs, we used a sparse imaging procedure with a repetition time (TR) of 8 s, including an effective acquisition time (TA) of 2 s interleaved with a silent gap of 6 s. Auditory stimuli were delivered binaurally via MRI-compatible headphones, and were presented with a varying jitter of 2.5 to 3.5 seconds relative to scan onset. For each participant, a high resolution T1-weighted anatomical scan was acquired using a TR of 9.6 seconds, TE of 4.6, FA of 8°, FOV 256 mm×256 mm×150 mm with 150 slices of 1.0 mm thickness.

### Image Processing

Functional images were preprocessed and analyzed with Statistical Parametric Mapping (SPM8; Wellcome Department of Imaging Neuroscience, London, UK, www.fil.ion.ucl.ac.uk) software implemented in MATLAB 7.8.0 (Mathworks Inc., Sherborn, MA). The first four images of each run were discarded to ensure signal stabilization. Images were realigned applying a rigid body spatial transformation of each of the BOLD volumes onto the fifth volume of the first run in order to remove movement artifacts. Functional images were co-registered with the anatomical scan and were stereotactically normalized into Montreal Neurological Institute (MNI) space on the basis of the structural T1-weighted 3D volume. Then, functional images were re-sliced at resolution of 3×3×3 mm and smoothed by a gaussian filter of 8×8×8 FWHM.

### Analysis

A statistical analysis on the basis of the general lineal model was performed using SPM8. In an event-related design, for each of the different emotional tones of voices as well as for the neutral stimuli, the expected hemodynamic response was modelled by the canonical hemodynamic response function (HRF; Friston et al., 1998) and its temporal derivative, as implemented in SPM8, with the silent trials serving as a baseline. Subsequently, parameter estimates of the HRF regressor for each of the different conditions were calculated from the least mean squares fit of the model to the time series. Parameters estimates for the temporal derivative were not further considered in any contrast.

The resulting contrast images were subjected to one sample t-tests subsequently explored at a threshold of p<0.005. Correction for multiple comparisons to p<0.05 was achieved using a cluster extent threshold procedure first described by Slotnick et al. [44]–[45]. As reported in a previous study [45], the cluster extent threshold procedure relies on the fact that given spurious activity or noise (voxel-wise type-I error), the probability of observing increasingly large (spatially contiguous) clusters of activity systematically decreases [45]. Therefore, the cluster extent threshold can be enforced to ensure an acceptable level of corrected cluster-wise Type I error. For an individual voxel Type I error of p<0.005, this procedure identified a cluster extent of 18 contiguous resampled voxels as necessary to correct for multiple voxel comparisons across the whole brain at P<0.05.

The main analysis is comparable to previous research in that it was performed without including pitch as a parametric modulator. However, in a further analysis, a new model employing a regressor reflecting the standardized estimates (Z scores) of pitch for each trial by emotional valence was employed. In this way, the effect of pitch on HRF amplitude between conditions is controlled for.

## Results

### Behavioural data

During both tasks all four response categories were discriminated significantly above chance level of 25% (simple emotion task [Mean accuracy ±simple emotion]: Happy: 73.92±0.01; Angry 77.25±0.01%; Sad: 71.21±0.01%; Neutral 70.90±0.01%. complex emotion task: Proud 53.45±0.01%; Guilty 55.23±0.01%; Bored 59.37±0.01% and Neutral 57.03±0.01%) (all t>21.3, p<0.0001). Then, data were collapsed across all simple emotions for the SE task and all complex emotions for the CE task. The reason for collapsing the data lies in the fact that this study was designed to investigate the average neural correlates of simple and complex emotion, independently of the specific emotional valences. A paired sample t-test showed that participants performed significantly more accurate (*t*(18) = 14.88 *p*<0.001) in the simple emotion (*M* = 73.93, *SE* = 0.01) run in comparison to the complex emotion run (*M* = 56.22, *SE* = 0.01)

To investigate whether stimuli of the simple and complex emotion conditions differ not only in the emotional complexity but also in low level acoustical features, simple and complex emotion conditions were also compared according to sound amplitude, duration and pitch mean. These acoustical features were extracted from the stimuli sound files using Praat software for the analysis of speech in phonetics available at http://www.fon.hum.uva.nl/praat/. Paired t-tests revealed no differences between conditions in amplitude, *t*(70) = 1.06 *p* = 0.295, and duration, *t*(70) = 0.83 *p* = 0.412. However, there was a significant difference in pitch between simple and complex emotion. The analysis revealed that simple emotion stimuli (M = 246.22, SD = 96.36) have an average higher pitch than complex emotion stimuli (M = 166.93, SD = 59.10, *t*(70) = 6.16 *p*<0.001).

### Functional imaging data

#### Emotional versus neutral trials

**Figure 1:**
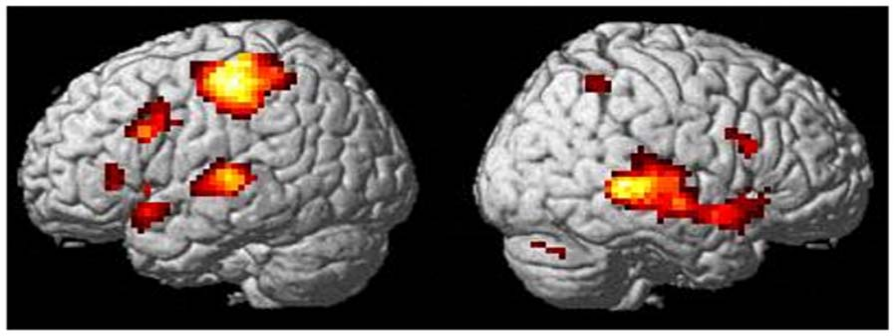
Regions involved in prosody for simple and complex emotion. Brain regions showing significantly stronger activations for simple and complex emotion as opposed to neutral trails. Activations are shown for p<0.05, corrected for multiple comparisons.

When neutral tone trials were compared to emotional trials across both runs (Table 1 and Figure 1), stronger activations for emotional as opposed to neutral trials were observed within the temporal lobe, in the middle and superior temporal gyri bilaterally, extending into the left temporal pole and the right insula. Within the frontal lobe, increased BOLD response was found in the inferior frontal operculum bilaterally and in the left pars triangularis. Additionally, the left precentral gyrus was activated. Further significantly activated clusters were observed in the right inferior parietal gyrus and right precuneus, the left putamen and the right cerebellum.

**Table 1:** Subtraction of neutral form emotional trials.

	structure	cluster size	Z score	*x*	*y*	*z*	BA peak
temporal lobe	R middle temporal	577	4.48	51	−37	1	21
	L superior temporal	222	4.14	−60	−22	4	22
	L superior temporal pole	40	3.49	−54	11	−14	38
	R insula	66	4.02	33	11	−8	48/38
frontal lobe	L precentral	633	4.82	−36	−28	61	4
	L inferior tri frontal	27	3.57	−36	32	1	47
	L inferior frontal operculum	119	3.46	−36	17	22	48
	R inferior frontal operculum	28	3.13	51	17	19	44
parietal lobe	R inferior parietal	33	3.38	33	−52	49	40
	R precuneous	30	3.27	12	−73	43	7
subcortical structures	L putamen	101	4.09	−24	17	−5	
	R cerebellum	30	3.29	33	−73	−22	

Local maxima of the brain regions activated more for simple and complex emotion as opposed to neutral trials at p<0.05 corrected for multiple comparisons. Coordinates refer to the MNI system.

#### Complex versus simple emotions

**Figure 2:**
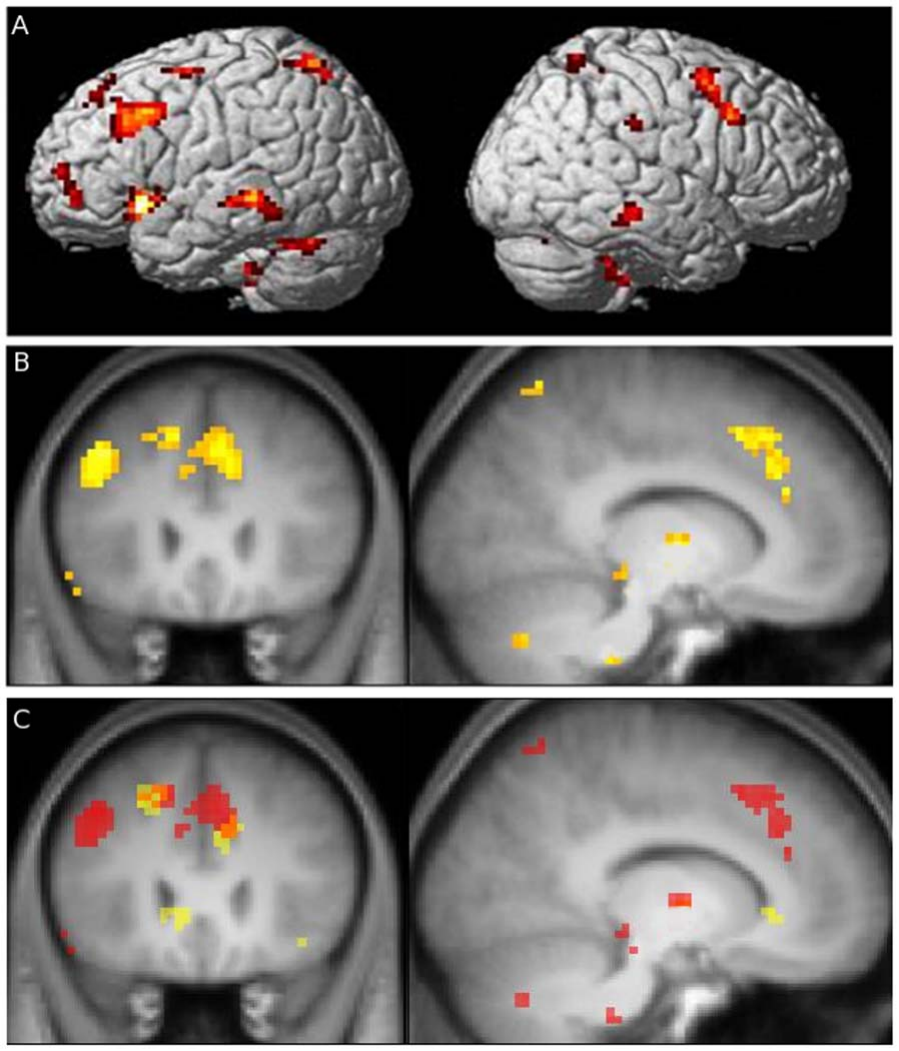
Regions involved in prosody for complex emotion. Brain regions showing significantly stronger activations for complex as opposed to simple emotion. The activation maps (at p<0.05, corrected for multiple comparisons) are shown overlaid onto a canonical brain rendered in three dimensions (A). The anatomical location of the medial frontal activation (at p<0.05, corrected for multiple comparisons) is shown overlaid onto the mean high-resolution T1 scan of the group (B). In (C) activations for the standard analysis are shown in yellow and activations corrected for confounding effects of pitch between conditions are shown in red with the overlap of the standard analysis and the analysis corrected for pitch shown in orange.

When brain activation during perception of simple emotion trials was subtracted from activation during perception of complex emotion trails, there was an increased BOLD response within the frontal lobe, where significantly activated clusters were located in the middle orbito-frontal cortex, right frontal operculum, left supplementary motor area and in the superior medial frontal gyrus (BA 9/32) (Table 2 and Fig. 2). Within the temporal lobes, an increase of activations was found in the right inferior temporal gyrus, the left superior temporal and left fusiform gyrus, the left insula and the right hippocampus. Further significant activations were observed bilaterally in somatosensory association cortex of the parietal lobes, the left thalamus and the right cerebellum.

**Table 2:** Subtraction of simple from complex emotion trials.

	structure	cluster size	Z score	*x*	*y*	*z*	BA peak
temporal lobe	L insula	129	3.78	−30	20	−11	38
	R parahippocampal cortex	42	3.73	27	2	−26	36
	R inferior temporal	26	3.43	51	−34	−17	20
	L middle temporal	77	3.3	−63	−31	−8	21
	L fusiform	163	3.29	−30	−43	−23	37
frontal lobe	L middle frontal	140	4.02	−42	20	40	44
	R frontal operculum	38	3.34	48	17	40	44
	L supperior medial frontal	299	3.32	−6	41	46	9/32
	R supplementary motor	73	3.24	15	5	58	6
	L middle orbito-frontal	33	3.23	−36	50	−5	47/10
	L precentral	19	3.17	−36	−1	61	6
parietal lobe	L superior parietal	44	3.46	−18	−61	67	7
	R superior parietal	25	3.3	21	−55	64	5
subcortical structures	Ltalamus	450	4.11	−6	−28	−17	
	cerebellum	320	3.62	12	−79	−32	

Local maxima of the brain regions activated more for complex as opposed to simple emotion at p<0.05 corrected for multiple comparisons. Coordinates refer to the MNI system.

The reverse contrast did not show any significantly increased activations for simple as compared to complex emotion.

#### Complex versus simple emotions controlled for pitch

Stimuli of the simple and complex emotion condition did not only differ in the emotional complexity but also in pitch, a basic acoustical feature. Therefore, pitch was added to the model as a parametric regressor of no interest in order to control for its confounding effect between conditions. On the whole, this analysis delineates a similar brain network of complex emotion processing as previously described. In comparison to the previous analysis, this analysis revealed activations in the right and left superior frontal gyrus (BA 9/32) extending towards medial regions, the left precentral gyrus, the left and right insula, the right parahippocampal gyrus, the left thalamus and the right cerebellum (Table 3 and Fig. 2C).

**Table 3:** Subtraction of simple from complex emotion trials (controlled for pitch).

	structure	cluster size	Z score	*x*	*y*	*z*	BA peak
temporal lobe	R parahippocampal	101	3.67	27	−25	−26	30/36
	R insula	20	3.51	33	17	−5	47/48
	L insula	30	3.3	−33	23	−11	47/48
frontal lobe	R (medial) supperior frontal	64	3.6	15	20	49	32/9
	L (medial) supperior frontal	36	3.47	−6	41	46	32/9
	R frontal middle	88	3.59	30	8	49	6
	L precentral	286	3.58	−36	−25	58	4/6
subcortical structures	R cerebellum	31	3.66	42	−52	−23	
	L thalamus	124	4	−9	−7	1	

Local maxima of the brain regions activated more for complex as compared to simple emotion controlled for confounding effects of pitch at p<0.05 corrected for multiple comparisons. Coordinates refer to the MNI system.

## Discussion

This study was conducted to reveal differences between the neural correlates of EPC for complex as opposed to simple emotion. Disentangling the brain representation for these different types of emotion should contribute to the prevailing controversies regarding the involvement of frontal brain regions in EPC.

### Perception of emotional versus neutral trials

In agreement with previous fMRI findings [19], [46]–[47], our data showed that EPC in general (pooling across simple and complex emotion relative to neutral trials), is supported by a temporo-frontal network, comprising the middle and superior temporal gyri, left temporal pole, right insula, Broca's area and its right hemisphere homologue, as well as the left motor cortex. Within this network, it is especially the right lateral temporal lobe and the right superior temporal gyrus (rSTG) that have been shown to be crucial for prosody decoding [48]–[50]. The additional involvement of left lateral temporal regions in the EPC task might not be related to EPC per se, but rather to explicit verbal labelling of emotional valences [51]. Interestingly, the middle temporal gyri, Broca's area and the left motor cortex have been linked to an auditory mirror neuron system identified in humans [52] and an activation of this network might be related to the empathic abilities of the individual [27], [52]. Thus, this activation might be associated to the processing of emotional tones.

After sensory evaluation of prosodic features in the STG, the output presumably is transferred towards more anterior regions for further processing, as it has been proposed by an analysis of effective connectivity [18]. Along the pathway towards anterior regions, an involvement of the left temporal pole in EPC was found. This paralimbic structure has been considered responsible for coupling visceral emotional responses with representation of complex auditory stimuli [53]. Another border structure between the temporal and frontal lobes in which EPC is processed is the insula. The results show right insula activation during EPC, interpreted as related to amalgamating interoception of body states and emotion [54].

Activations in Broca's area and its homotopic region during EPC are in line with several previous neuroimaging studies [21], [27], [55]. An involvement of the right IFG has been suggested for explicit evaluative judgements of emotional prosody, whereas the activation of the left IFG may reflect integration of vocal and verbal information [56]. Further activation of the frontal lobe was found in the left precentral gyrus. This activity should not be related to the motor response (button pressing) because such activation should have been identical for emotional and neutral trials. An alternative interpretation of the motor activation triggered by emotional stimuli relates to a preparation of motor responses to perceived emotion, such as the mimic of a communicative gesture to respond to the perceived emotion [28].

Finally, the activation of the right inferior parietal gyrus and precuneus is consistent with previous findings showing a role of this region in explicit emotional stimuli, as compared to phonetic/semantic stimuli [22], [55]. This activation has been interpreted as higher order analyses of auditory signals [55] in polymodal areas of the parietal cortex.

### Perception of complex versus simple emotions

Although contradictions exist [18], [57], some previous studies found the mPFC to be involved in emotional prosody [58]–[59]. Our key finding revealed that the same regions of mPFC reported in previous studies [58]–[59] were activated specifically for the complex emotion task, suggesting that the mPFC is one of the key structures for decoding complex emotion. Moreover, an additional analysis, controlling for the effect of pitch between condition still reveals mPFC activation. This analysis has been performed because it has been shown that pitch perception, a low level sensory process, correlates with EPC at least at the behavioural level [60]. Thus, EPC brain representation might be confounded by pitch. The present result did not find evidence that mPFC role in EPC is confounded by pitch, in spite of its connection to temporal regions processing this low level sensory property. Instead, the involvement of the mPFC presumably reflects higher cognitive processes intrinsic to complex emotion such as inferring other's mental states. Given that the mPFC is involved in social cognitive processes, such as recognition of conspecifics and understanding of other's emotions intentions and beliefs [35], it is likely that the recruitment of mPFC in EPC tasks depends on the extent to which participants engage with another's perspective when attempting to decode their emotions. Decoding non-social complex emotions is based on the interpretation of cognitive beliefs which caused the current mental state, and decoding complex social emotion require the interpretation of social intentions. The mPFC activation underlies the interpretation of cognitive beliefs and social intentions, which are necessary for complex emotion, but only contingent to comprehend simple emotion.

The mPFC activation in the EPC task may be related to ToM strategies applied to the comprehension of complex emotion. In order to interpret social complex emotions, individuals may need to simulate the feelings of other people in their own mind in order to understand them. In accordance with this finding, the mPFC has been particularly associated to understanding intentions of others [61], affective evaluation of imagined objects [62], and it is also a component of the network supporting modality independent emotion perception [63]. Moreover, recruitment of this region was found in emotional speech comprehension, and it has been interpreted as related to inferring and sharing other's emotion [58].

Alternatively it could be argued that the mPFC activation is due to increased task demands during complex emotion as compared to simple emotion perception. As has been said, the acoustical features of prosody for complex emotion are less differentiated; thus processing EPC for complex emotion strongly relies upon cognitive interpretation, in detriment of perceptual processing resulting in a greater degree of uncertainty. This would be in line with the behavioural data showing a higher accuracy for simple emotion in comparison to complex emotion trials. In line with this interpretation, a model of error likelihood postulated by Brown and Braver [64] proposes that the anterior cingulate and mPFC play a role in predicting the probability of an error to occur. Although the greater likelihood for an error to occur in EPC for complex emotion might increase the recruitment of mPFC, a model of prefrontal function [65] has demonstrated that cognitively more demanding tasks rather rely on the recruitment of more dorso-lateral regions of PFC. Following this line of argument, an increase in cognitive demands causing mPFC recruitment in complex emotion should be less likely. Moreover, mPFC seems to be rather involved in low demand situations, such as the absence of a task requiring deliberative processing as it has been included in the brain default mode network [66]. Although dualist interpretation regarding the behavioural correlates of the default mode network should be taken cautiously, it has been proposed that when people are at rest, they might mentally project themselves to imagine the viewpoint of others, an activity similar to ToM [67]. Thus, the observed mPFC activity might reflect the involvement of ToM strategies while solving EPC for complex emotion.

Noteworthy, the mPFC might be necessary but not sufficient for ToM. In addition to the mPFC, the present study revealed activation in the supplementary motor area as well as in the somatosensory association cortex, which is in line with the mirror neuron system role in ToM. In fact, ToM tasks focussed on indentifying beliefs and emotional states have shown to recruit the mPFC, the IFG and somatosensory association cortex [68], which was interpreted as the use of internal affective representations to understand other's emotions [68].

Besides the somatosensory and premotor cortices, the right frontal operculum and insula were additionally recruited for complex and social in comparison to simple emotion. However, these structures might be involved in differential aspects of EPC for complex emotion. The somatosensory and premotor cortex form a modality independent representation of emotions [69] and it has been shown that these regions are needed for the processing of facial [70] as well as vocal affect [28], [71], meaning that they form part of a general mirror neuron system for emotion processing. In contrast to the multimodal representation of emotion in somatosensory and premotor cortex, the right inferior frontal operculum seems to be related with the processing of vocal emotion in particular. The right frontal operculum, a part of the audio motor loop, comprises the engrams of orofacial movements necessary for an automatic motor mapping of prosody [72]. Interestingly, more empathic individuals recruit motor regions to a greater extent during EPC tasks [27] thus, the recruitment of the Broca's area homologue during complex emotion perception might be driven by the increased need of empathizing during complex emotion comprehension. Interestingly, Broca's homologue was also present in the subtraction of neutral from emotion trials (simple and complex together) probably because the complex trials drove this region activation. Finally, the anterior insula, known for linking the perception of emotional stimuli with visceral responses, would be involved in sensing one's own bodily state [73]. It is likely that the somatosensory and premotor cortex simulate the perceived emotion, the anterior insula adds visceral reaction, the inferior frontal gyrus activates orofacial movements egrams respond to the perceived emotion and the mPFC disentangles one's owns mental states from those of others.

Complex emotion comprehension also revealed activations within the temporal lobe, such as the left middle temporal gyrus. The right middle temporal gyrus has a role in prosody decoding [18], [57]. The left lateralized response of this region might be related to the more linguistic aspects of the stimuli. Other temporal activations, such as those of the right parahippocampal cortex and fusiform area are in agreement with a study of multimodal emotion perception in which medial temporal regions are triggered by the amygdala in the presence of emotionally salience stimuli [74]. Medial temporal regions process memory for emotional arousing material automatically, being a gate between emotion and cognition [74].

As a final consideration, it is noteworthy that the subtraction of complex from simple emotions (simple minus complex) did not reveal any activation. This null finding indicates that simple emotion does not involve any cognitive perceptual process not conveyed by complex emotion.

In sum, the present study revealed that EPC for complex emotion and for simple emotion share the same emotional-perceptual network. However, additional social and cognitive neural components are recruited when processing complex emotion. By controlling for pitch differences between conditions, the present study suggests that prefrontal involvement in EPC for complex emotion might be relatively independent of low level acoustical features. Key structures as mPFC and rSTG, and somatosensory association cortex are crucial for EPC of complex emotions. This neural network is very similar to the network that has been found in studies focussing on ToM. It is possible that inconsistent involvement of the mPFC as well as the somatosensory cortex in EPC is due to the extent in which participants try to infer belief and intention of external agents. Since making inferences about social intentions and mental states for the comprehension for simple emotions is plausible but not necessary, this skill is essential for the comprehension of complex emotions.
